# Evaluating Practice Patterns of Observation Periods Following Epinephrine Administration for Anaphylaxis Among Pediatric Patients

**DOI:** 10.7759/cureus.69419

**Published:** 2024-09-14

**Authors:** Hannah B Short, Benjamin Walters, Maria Fabi, Nigel Ravida, Susan Boehmer, Lilia Reyes

**Affiliations:** 1 Department of Emergency Medicine, Penn State College of Medicine, Hershey, USA; 2 Department of Biostatistics, Penn State College of Medicine, Hershey, USA; 3 Department of Pediatric Emergency Medicine, Penn State Health Milton S. Hershey Medical Center, Hershey, USA

**Keywords:** anaphylaxis, biphasic reaction, epinephrine, observation period, pediatric

## Abstract

Objective

Current guidelines recommend that anaphylactic patients be observed for 4-6 hours following epinephrine administration to monitor for biphasic reactions. There is conflicting data regarding the efficacy of these guidelines and the prevalence of biphasic reactions among the pediatric population. This retrospective study aimed to investigate the appropriateness of these guidelines through evaluation of observation periods and patterns of biphasic reaction development among pediatric anaphylactic patients at a single-institution ED.

Methods

Patients less than 18 years of age who presented to the ED of a tertiary academic medical center between 2017 and 2022 and were treated with epinephrine for anaphylaxis were included in the study. The frequency and timing of biphasic reactions were observed. Duration of ED observation, time between symptom onset and first dose of epinephrine, number and category of anaphylactic symptoms, and allergen type were compared between patients who did and did not experience a biphasic reaction. Additional variables analyzed included persistence of anaphylactic symptoms, additional doses of epinephrine, and adjuvant medications. Contingency tables and two-sample t-tests were used to compare categorical and continuous variables, respectively, between those who did and did not develop a biphasic reaction.

Results

A total of 292 patients met the inclusion criteria and were included in the analysis. All patients were observed in the ED for a mean of 233.1 minutes. Ten patients (3.4%) developed a biphasic reaction. Six had a reaction within 150 minutes of initial symptom resolution, and four developed one after discharge, within 10 to 33 hours following symptom resolution. There was no significant difference in the length of time observed in the ED (p=0.98) or from symptom onset to the first epinephrine dose (p=0.90) between groups. Presenting with respiratory symptoms was associated with persistent anaphylactic symptoms despite epinephrine administration (p=0.01). Patients with symptoms involving at least two organ systems were 3.45 times more likely to experience persistent symptoms post-epinephrine than those with involvement of only one organ system (OR=3.45; CI: 1.28-9.30). Allergen type, anaphylactic symptoms, or the number of organ systems involved were not linked to developing a biphasic reaction or the need for additional epinephrine doses.

Conclusions

The patients who developed a biphasic reaction did so either shortly following initial symptom resolution or many hours past the recommended observation period. Extending the observation period of patients within reasonable parameters would not have reduced the number of patients who experienced a biphasic reaction after discharge. The results of this study support potentially adopting a more individualized approach to anaphylaxis management following epinephrine administration. Shortening the observation period for patients at low risk for biphasic reactions could reduce the patient burden on EDs without negatively impacting patient outcomes. Although no significant risk factors for biphasic reactions were identified in this study, closer monitoring of patients with respiratory symptoms and/or involvement of a greater number of organ systems may help mitigate the number of patients who experience persistence of anaphylaxis despite treatment.

## Introduction

Anaphylaxis is an acute and systemic allergic reaction that can compromise the airway, circulatory, and respiratory systems, and can potentially result in death [1,2]. It is a type I hypersensitivity reaction, meaning it is primarily mediated by IgE [1]. IgE, an immunoglobulin associated with allergic reactions and parasitic infections, targets a specific allergen surface antigen it has previously come into contact with. Upon re-exposure to an antigen, the pre-formed IgE binds to both the surface antigen and the mast cell IgE receptor, triggering mast cell degranulation. Activated mast cells release pre-formed inflammatory mediators, including histamine, leukotrienes, cytokines, and prostaglandins, that recruit additional inflammatory cells, propagating the inflammatory reaction. The immediate release of these signaling molecules upon allergen exposure is responsible for both the acute onset of symptoms and the widespread involvement of multiple organ systems [1].

The United States National Institute of Allergy and Infectious Diseases (NIAID) sets forth guidelines for both the diagnosis and management of anaphylaxis. Anaphylaxis is a clinical diagnosis described as the involvement of at least two organ systems, including respiratory (stridor, wheezing, and bronchoconstriction), cardiovascular (tachycardia, hypotension, and syncope), gastrointestinal (nausea, vomiting, and diarrhea), and/or mucosal (flushing, urticaria, angioedema, and pruritus) [1,2]. Exposure to a known trigger may or may not be necessary for the fulfillment of criteria, as there are three domains that can be used for diagnosis. In the pediatric population, anaphylaxis is most commonly caused by food-related allergens, with peanuts, tree nuts, and dairy being the most common culprits [1]. Other common triggers include stinging insect venom and medications, including antibiotics and non-steroidal anti-inflammatory drugs (NSAIDs) [3].

The first-line treatment for anaphylaxis among both adult and pediatric patients is an intramuscular injection of epinephrine. Epinephrine primarily acts as an agonist on the β1, β2, and α1 adrenergic receptors [4]. This results in a variety of physiologic responses that counteract the effects of inflammatory mediators, including vasoconstriction, bronchodilation, and decreased gastric motility. Epinephrine has a short half-life of 2-3 minutes due to rapid enzymatic degradation and reaches peak plasma concentration by intramuscular injection within 5-10 minutes [4,5]. Delayed administration of epinephrine for anaphylaxis is associated with an increased need for hospitalization, poorer outcomes, and death, reinforcing its therapeutic effects and the importance of prompt administration [6]. Among pediatric patients, the NIAID recommends administration of 0.01 mg/kg of a 1 mg/mL solution intramuscularly. Recurrent or unresponsive symptoms can be followed by additional doses, not exceeding 0.3 mg in a pre-pubertal child and 0.5 mg in an adolescent [6]. Adjuvant medications for the treatment of anaphylaxis include H1-blockers, H2-blockers, beta-2 adrenergic agonists, and corticosteroids. These medications are effective in reducing various symptoms of anaphylaxis; however, they should not be used for acute treatment, as their effects are significantly inferior to those of epinephrine [7,8].

In some cases, anaphylactic symptoms may spontaneously recur after complete resolution of symptoms following treatment. This is known as a biphasic reaction and can occur up to 72 hours after the initial onset of symptoms [9]. These reactions are often attributed to a second wave of mast cell degranulation, uneven absorption of the offending allergen, or reactivation following treatment [4]. In pediatric populations, the incidence of biphasic reactions occurs in 5% to 20% of anaphylaxis cases, with most studies reporting their occurrence, on average, over eight hours after the initial resolution of symptoms [1]. Developing a biphasic reaction has been associated with several factors, including the requirement for multiple doses of epinephrine, delayed administration of epinephrine, delayed presentation to the ED, and the need for additional therapeutic interventions, such as inhaled bronchodilators for respiratory distress [5]. Anaphylaxis onset manifesting with hypotension and shock has been linked to biphasic reactions, supported by many studies over the past few decades [10].

Currently, guidelines are inconsistent in dictating the appropriate observation time in the ED after the administration of epinephrine for pediatric anaphylaxis cases. While the NIAID recommends observation for 4-6 hours following epinephrine administration, some studies cite observation times of up to 24 hours, and others as little as one hour [11,12]. In fact, a majority of biphasic reactions in the literature occur outside the 4-6 hour observation period, questioning the utility of this guideline [12]. The average duration of time that pediatric anaphylaxis patients are observed in the ED following epinephrine administration is not well defined. As EDs face increasing patient demand with concurrent limited resources and space, long observation periods post-anaphylaxis may crowd the already limited hospital space. It is therefore important to further characterize the observation patterns of EDs for post-epinephrine pediatric anaphylaxis patients and evaluate additional factors that could help predict a patient’s likelihood of developing a biphasic reaction.

The primary objective of this study was to investigate the appropriateness of NIAID guidelines for the observation of anaphylactic pediatric patients post-epinephrine administration through the evaluation of patterns of observation time and biphasic reaction onset at a single-institution ED. Secondary objectives included determining the prevalence of biphasic reactions and factors that may be associated with the development of a biphasic reaction, including the number and type of presenting anaphylactic symptoms, delay in epinephrine administration, and allergen type.

## Materials and methods

Study population

The ICD-10 codes (T78.2: Anaphylaxis, unspecified; T78.2XXA: Anaphylactic Shock; T78.0: Anaphylaxis from food; W57.XXXA: Insect bite; L50.0: Urticaria; T78.40XA: Unspecified allergic reaction) were utilized to identify patients who presented to the pediatric emergency department (PED) of a tertiary academic medical center between 2017 and 2022 for anaphylaxis. Patients were eligible for inclusion in the study if they were younger than 18 years at the time of presentation, presented to the PED with a presumed anaphylactic reaction, and received therapeutic intramuscular epinephrine either prior to arrival or while in the ED. Individuals who did not meet the above inclusion criteria were excluded from the study. The study was approved and deemed exempt by the Penn State Institutional Review Board (IRB).

Outcome variables

The times at which patients received their first dose of intramuscular epinephrine, arrived at the ED, and were discharged from the hospital were recorded. These time points were used to calculate the total observation time in the ED and the length of time between the onset of anaphylaxis and the first dose of epinephrine. The observation period post-epinephrine administration concluded if the patient was admitted to the hospital or sent to the ED observation unit. The frequency of biphasic reactions was recorded for patients, defined by the return of anaphylactic symptoms within 72 hours after epinephrine administration. Any subsequent doses of epinephrine were noted, in addition to the persistence of anaphylactic symptoms, defined by a lack of clinical improvement after a dose of epinephrine. In patients who received epinephrine prior to hospital arrival, anaphylactic symptoms were defined as the subjective symptoms reported by the patient or caregiver. Objective symptoms documented in the medical record as vital signs or clinical exam findings were utilized for patients who received their first epinephrine dose in the ED or pre-hospital by EMS. Symptoms were then categorized by system involvement, including cardiac, gastrointestinal, mucosal, and respiratory. Lastly, the causative allergen was noted, in addition to adjuvant medications provided either prior to or in the hospital.

Statistical analysis

Descriptive statistics were generated, including means, medians, SDs, and confidence intervals for continuous variables; frequency tables and percentages were calculated for categorical variables. Differences between groups for categorical variables were characterized using contingency table analysis. Significance levels were determined by Pearson’s chi-square statistic and Fisher’s Exact Test. Differences between groups for continuous variables were determined by two-sample t-tests. P-values less than 0.05 were considered significant. The statistical analysis was performed using SAS software, version 9.4.

## Results

Of the 1,017 patients identified using ICD-10 codes, 292 met the inclusion criteria and were included in the study. Of those patients, 118 (40.4%) were biological females and 174 (59.6%) were biological males. The mean age at the time of presentation was 8.8 years (SD = 5.7). Demographic variables of the study population are displayed in Table 1.

**Table 1:** Study population demographics.

Total patients (n=292)		
Age (years), mean (SD)	8.8 (5.7)	
Sex, n (%)	Female	118 (40.4%)
	Male	174 (59.6%)
Age, n (%)	< 1 year	23 (7.9%)
	1-5 years	78 (26.7%)
	6-10 years	58 (19.9%)
	11-13 years	44 (15.1%)
	14-17 years	89 (30.5%)

A total of 207 patients (70.9%) presented with an anaphylactic reaction secondary to food, 22 (7.5%) to medication, 14 (4.8%) to insect bites, 2 (0.7%) to animals, 2 (0.7%) to plants, and 45 (15.4%) to an unknown source or one that did not fit the previous categories. A total of 129 patients (44.2%) received epinephrine prior to arrival at the ED, and the remaining 163 (55.8%) received the initial dose upon arrival. The mean time between the onset of anaphylactic symptoms and the first dose of epinephrine was 93.9 minutes (CI 80.0-107.8, SD = 120.0). On initial presentation of anaphylaxis, 39 patients (13.4%) experienced cardiac symptoms, 122 (41.8%) respiratory, 265 (90.8%) mucosal, and 105 (36.0%) GI. Among all 292 patients, there was an average of 1.8 symptom categories present at the onset of anaphylaxis (CI 1.7-1.9, SD = 0.8). A total of 180 patients (61.6%) presented with symptoms involving at least two organ systems, and the remaining 112 patients (38.4%) presented with symptoms involving only one organ system. Of these 112 patients, 87.5% (n=98) presented with only mucosal involvement. While in the ED, 59.6% of patients (n=174) received diphenhydramine, 43.5% (n=127) famotidine, 33.6% (n=98) methylprednisolone, 12.3% (n=36) prednisone, 47.6% (n=139) dexamethasone, 11.3% (n=33) albuterol, 5.1% (n=15) DuoNeb, and 7.2% (n=21) ondansetron for adjuvant therapy.

Primary outcomes

**Figure 1:**
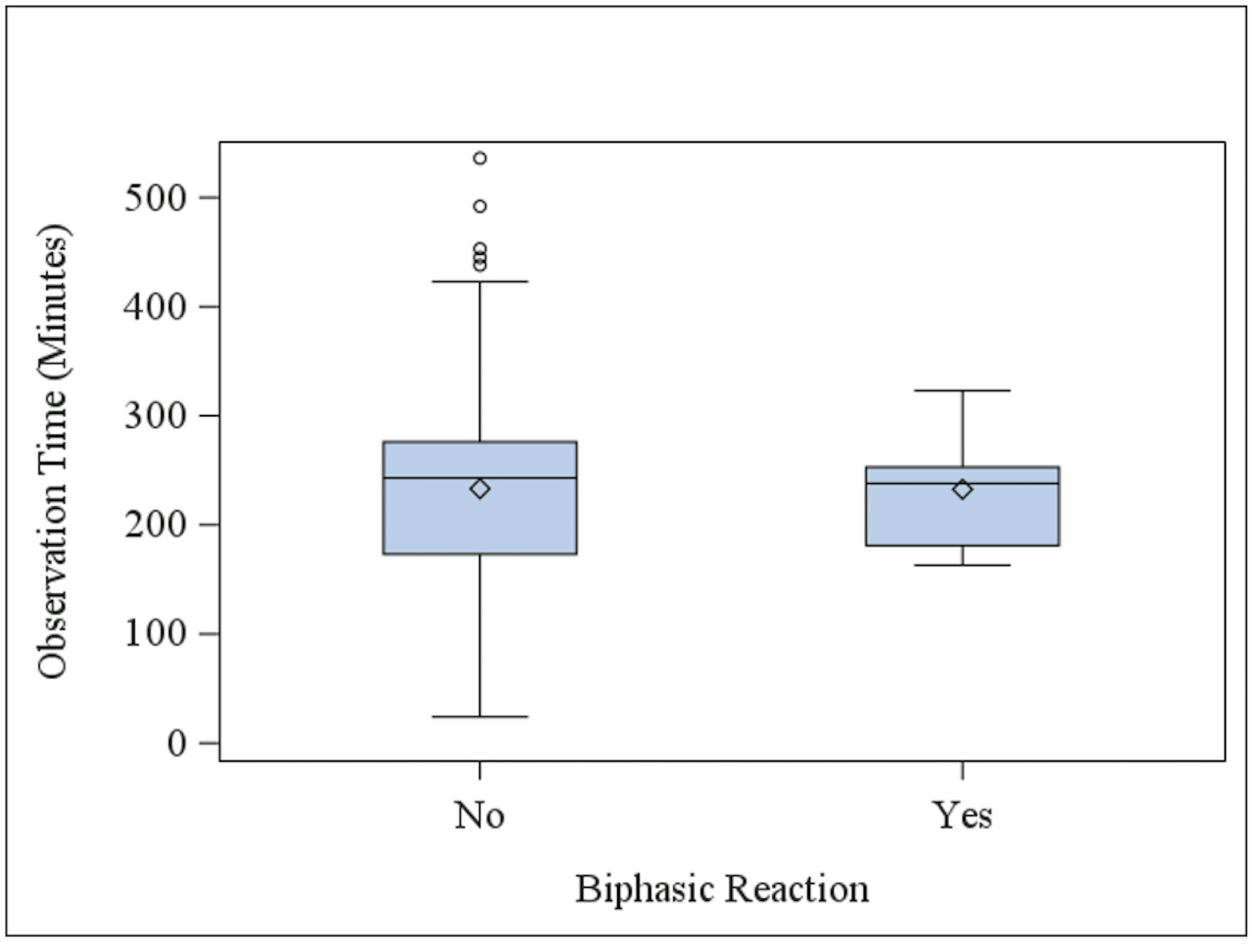
Post-epinephrine observation time in the ED. The observation periods (in minutes) of patients after epinephrine administration are displayed for those who did and did not experience a biphasic reaction. Patients who experienced a biphasic reaction were observed for an average of 232.6 minutes, while those who did not were observed for an average of 233.2 minutes (p = 0.98).

All patients were observed in the ED after epinephrine administration for a mean of 233.1 minutes (CI 224.0-242.3, SD = 78.9). Patients who experienced a biphasic reaction were monitored in the ED for an average of 232.6 minutes (CI 197.6-267.6, SD = 48.9), while those who did not were monitored for an average of 233.2 minutes (CI 223.8-242.5, SD = 79.8), as shown in Figure 1 (p = 0.98).

Of the 292 pediatric patients with anaphylaxis, 3.4% (n=10) experienced a biphasic reaction, and the remaining 96.6% (n=282) did not. The mean age of patients who did and did not develop a biphasic reaction was 11.1 (SD=5.1) years and 8.7 (SD=5.8) years, respectively. There was no significant difference in mean age between groups (p = 0.20). Four of the ten patients (40.0%) developed a biphasic reaction after discharge from the hospital. Among these four patients, a biphasic reaction occurred between 10 to 33 hours after resolution of initial symptoms and required re-presentation to the ED. The remaining six patients experienced a biphasic reaction in the ED within an average of 96.3 minutes after initial symptom resolution, with values ranging between 30 minutes to 150 minutes.

**Figure 2:**
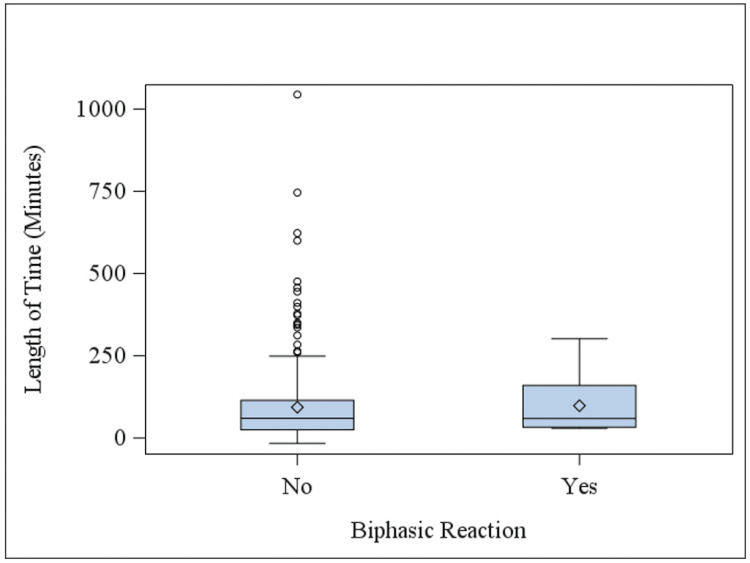
Time period between anaphylaxis onset and first dose of epinephrine. The time (in minutes) between the onset of anaphylactic symptoms and the first dose of intramuscular epinephrine is displayed for those who did and did not experience a biphasic reaction. Patients who developed a biphasic reaction received their first dose of epinephrine an average of 98.4 minutes after the onset of symptoms, compared to an average of 93.7 minutes among those who did not (p = 0.90).

Figure 2 displays the average time between onset of anaphylactic symptoms and the first dose of epinephrine between those who experienced a biphasic reaction and those who did not. Individuals who experienced a biphasic reaction had a non-significantly higher mean of 98.4 minutes (CI 35.7-161.1, SD = 87.6) compared to the mean of 93.7 minutes (CI 79.4-108.1, SD = 121.0) among those without (p = 0.90).

Among the 10 patients with a biphasic reaction, 70.0% (n=7) experienced an anaphylactic reaction from food, 10.0% (n=1) from medication, and 20.0% (n=2) from an additional/unknown category. 70.9% of patients (n=200) who did not experience a biphasic reaction had anaphylaxis secondary to food, 0.7% (n=2) from animal species, 5.0% (n=14) from insect, 0.7% (n=2) from plant, 7.5% (n=21) from medication, and 15.3% (n=43) from an additional/unknown category. There was no significant relationship between the allergen type and the development of a biphasic reaction (p = 0.97).

**Figure 3:**
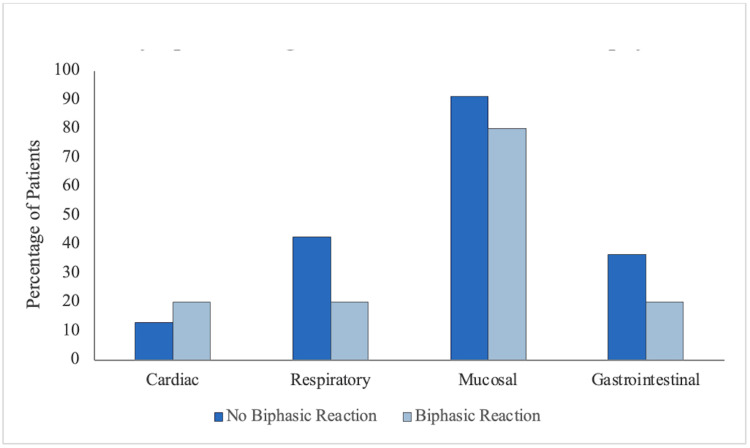
Symptom categories present at onset of anaphylaxis. Bars represent the percentage of patients who presented with cardiac, respiratory, mucosal, and gastrointestinal symptoms at the onset of anaphylaxis.

Figure 3 exhibits the percentage of biphasic and non-biphasic reaction patients who experienced cardiac, respiratory, mucosal, and GI symptoms at the onset of the initial episode of anaphylaxis. There was no significant relationship between experiencing a biphasic reaction and presenting with cardiac (p = 0.53), mucosal (p = 0.23), respiratory (p = 0.16), or GI symptoms (p = 0.28).

There was no significant difference in the mean number of symptom categories present at the onset of anaphylaxis between biphasic groups, with biphasic patients experiencing an average of 1.4 (CI: 1.0-1.8, SD = 0.5) symptoms and non-biphasic patients 1.8 (CI: 1.7-1.9, SD = 0.8) symptoms (p = 0.38).

Secondary outcomes

There was no significant relationship between allergen type and presenting with cardiac (p = 0.89), respiratory (p = 0.35), or mucosal symptoms (p = 0.94). Experiencing GI symptoms was significantly associated with the category of allergen (p = 0.0003). A total of 33 (11.3%) patients required at least one additional dose of epinephrine, with a mean age of 11.7 (SD=4.9) years. This was significantly higher than the mean age of patients (8.4 years, SD=5.7) that did not require additional epinephrine doses (p = 0.002). Need for an additional dose of epinephrine was not significantly associated with allergen type (p = 0.96) or number of symptom categories at anaphylaxis onset (p = 0.55). Additionally, associations remained statistically insignificant for cardiac (p = 0.82), respiratory (p = 0.65), mucosal (p = 0.55), and GI symptoms (p = 0.06). A total of 30 patients (10.3%) experienced persistence of anaphylaxis symptoms after the therapeutic action of epinephrine. Persistence of symptoms was not significantly associated with allergen type (p = 0.65) or cardiac (p = 0.57), mucosal (p = 0.88), or GI (p = 0.93) symptoms. Individuals who presented with respiratory symptoms at anaphylaxis onset were significantly more likely to experience persistence of symptoms after epinephrine administration (p = 0.01). Additionally, those who experienced three or more symptom categories were 3.45 more likely to experience persistence of symptoms compared to those who experienced one symptom category (OR=3.45; CI 1.28-9.30).

## Discussion

In this study, it was found that the average time observed in the ED following epinephrine administration was slightly shorter than the recommended 4-6 hours set forth by the NIAID. There is considerable variation in observation periods proposed in the literature, reflecting the inconsistent pattern of biphasic reaction onset observed across studies [11,12]. As a result, recommendations are transitioning towards a more individualized management plan [13]; in the absence of persistent or refractory symptoms of anaphylaxis, a patient’s observation period may be determined using clinical judgment (patient risk factors, clinical presentation) in conjunction with national guidelines [13]. Therefore, while the observed periods in this study were noteworthy in terms of local institution practice patterns, they have limited clinical value when analyzed alone. What is arguably more clinically impactful is when biphasic reactions occurred and how this compares to both the national guidelines and the patterns of observation times of the associated institution.

The frequency with which biphasic reactions occurred was lower than anticipated. Biphasic reactions among the pediatric population are reported to occur in 5 to 20% of anaphylactic patients [1], and the results of this study fall outside of this range. The frequency reported in studies varies widely, and it is likely that this and other studies' patient population, study design, and definition of a biphasic reaction have a large impact on these noted variations [10]. Among all the patients who developed a biphasic reaction, the time between initial anaphylaxis resolution and biphasic reaction onset was either early in their clinical course or delayed far outside the period of national guidelines. The patients who developed a biphasic reaction after discharge from the ED did so at a minimum of 10 hours following initial symptom resolution. This observation is consistent with findings from previous studies; in fact, a meta-analysis of 2,890 adults with anaphylaxis found a median time of 10.5 hours (range of 1.75 to 17 hours) between anaphylaxis resolution and biphasic reaction onset [14]. Although this study was limited to adults, other studies limited to or inclusive of pediatric patients have reported comparable results. For instance, a 2014 systematic review of both adult and pediatric anaphylactic patients reported a mean length of time of 8.13 hours between initial symptom resolution and biphasic onset [12]. Extending the observation period to 24 hours would not have benefited all these patients, and doing so for all anaphylactic patients would likely result in misuse of critical ED resources, outweighing the potential benefit it may provide. Identifying clinical predictors that could aid in selectively prolonging observation periods for patients would be more useful. Although the group sizes were too small to determine if differences existed between biphasic groups who developed recurrence of symptoms early on versus late in their clinical course, this would be a compelling topic of investigation for future studies. In addition, predictors of biphasic reactions in general may be useful in determining which patients should be observed for longer periods of time.

Widened pulse pressure, hypotension and/or shock, the need for multiple doses of epinephrine, delayed epinephrine administration, and increased anaphylaxis severity have been associated with the development of biphasic reactions [5,14]. Our study did not find an association between cardiac symptoms and biphasic reactions, which is an association cited in many previous studies [5]. It is possible that cardiac symptoms were less likely to be reported by guardians compared to other symptoms, as they may be less easily identifiable on visual examination. In addition, a limitation of the retrospective nature of this study is the potential for initial epinephrine administration to mask cardiac symptoms more rapidly than other symptoms, ultimately preventing identification on exam and subsequent documentation in patient charts. Cardiac symptoms occur less frequently than those of other organ systems in anaphylactic reactions [15], so the frequency observed in this study could reflect this with or without a degree of underreporting, as described. Likewise, our results also did not demonstrate an association between delayed epinephrine administration and biphasic reactions. Our study population may have been too small to detect this difference. For instance, a 2015 retrospective review identified delayed epinephrine administration as a risk factor for later development of a biphasic reaction, with 71 of 484 pediatric patients doing so [10]. This observation has been found in multiple other studies as well [16-18]. Therefore, prompt administration of epinephrine for anaphylaxis should remain a priority for healthcare providers, patients, and patient guardians.

Severity of the initial anaphylactic reaction has been associated with the development of a biphasic reaction [12]. Our study found that persistence of symptoms, rather than biphasic reactions, was associated with the number of symptom categories present at anaphylaxis onset. This suggests that identifying anaphylaxis severity through the number of organ symptoms involved may provide useful information on which patients may need closer initial monitoring and/or more aggressive treatment with medication. Interestingly, it was also found that respiratory symptoms were associated with persistence of symptoms, however not with biphasic reaction. Multiple studies have identified respiratory involvement as a predictor of lack of development of biphasic reaction [19], however, evidence on the association with persistence of symptoms is lacking. Additional predictors of biphasic reaction previous studies have identified include the need for additional doses of epinephrine [10] and anaphylaxis trigger [3,14], neither of which were found to have an association in this study.

A majority of biphasic reactions occur outside the recommended observation period [12]. Our study found that nearly half of those who experienced a biphasic reaction did so well after the nationally recommended ED observation time. Given the extremely high patient volumes in EDs, limiting the misuse of medical resources and hospital beds has become increasingly important. This questions the practice of observing all anaphylaxis patients treated with epinephrine for the recommended 4-6 hours. Based on the patients in this study, shortening this period to two hours, for instance, would have had no impact on the number of patients discharged from the ED before a biphasic reaction developed. The findings support adopting a more individualized approach to anaphylaxis management by shortening the observation period for patients who are not at increased risk of a biphasic reaction. Unfortunately, this study did not identify patient risk factors that increase the likelihood of biphasic reactions, although it found several that may help predict the persistence of symptoms, rather than their recurrence.

Limitations and future directions

A limitation of this study is its retrospective nature and that it was conducted at a single institution. Analyzing practice patterns at one institution introduces potentially unforeseen variables, such as institution-specific practices, that may impact the generalizability of the results. Given the aim was to investigate the appropriateness of national guidelines regarding the observation period of anaphylaxis patients following epinephrine administration, it is crucial for data to be representative of diverse patient populations. Future retrospective studies should include data from multiple institutions and geographic regions, not just a single location.

Obtaining accurate histories for patients who received epinephrine prior to hospital arrival largely depended on accurate recall by guardians. Symptoms such as hypotension or mild wheezing could be too subtle for identification and therefore not reported. Additionally, the administration of epinephrine might have masked initial cardiovascular manifestations of anaphylaxis, such as hypotension, which are more difficult to identify without specific medical tools. Therefore, these symptoms may have been more likely to be underreported due to their subtlety and rapid resolution after epinephrine administration. Future studies that gather data prospectively may avoid some of these drawbacks.

While medical records indicated when adjuvant medications were ordered, they may not have accurately or consistently provided the time they were administered. Adjuvant medications offer limited benefit in the acute treatment of anaphylaxis [20]; however, their timing may play an important role in clinical outcomes, such as the development of biphasic reactions. For instance, there is a reduced risk of prolonged hospitalization among children with anaphylaxis who receive glucocorticoids [21]. The timing of administration relative to symptom onset or epinephrine dose may impact this relationship and was not explored in this study due to the limitations described. An additional consideration for future studies is to stratify biphasic reactions based on symptom severity. Not all patients who developed a biphasic reaction experienced severe symptoms, and this may be a clinically important outcome to analyze, rather than considering biphasic reactions as a whole.

## Conclusions

The national guidelines for observation in the ED following epinephrine administration for anaphylaxis recommend a period of 4 to 6 hours. In this study, over half of the patients who developed a biphasic reaction did so within 150 minutes of symptom resolution, at least an hour and a half before the minimum recommended observation period. The remaining patients developed a recurrence of symptoms between 10 and 33 hours after initial symptom resolution, a finding consistent with previous studies. These results support transitioning to a more personalized approach to patient management, potentially shortening the observation period for patients deemed low risk for developing a biphasic reaction. This practice could minimize the burden on emergency departments without negatively impacting patient outcomes. Additionally, patient factors such as the number of organ systems involved and respiratory involvement may be useful for predicting the persistence of anaphylactic symptoms following treatment. Although this study did not identify significant risk factors for a biphasic reaction, such factors have been identified in previous studies. Future studies should seek to include a larger and more representative study population across multiple institutions and geographic regions.
